# Clinicopathologic characteristics of young patients with lip squamous cell carcinoma: a retrospective study

**DOI:** 10.4317/medoral.26740

**Published:** 2024-10-13

**Authors:** Pedro Augusto Bulhões Curioso, Ivan José Correia-Neto, Lucas Lacerda de Souza, Edilmar de Moura Santos, Pablo Agustin Vargas, Alan Roger Santos-Silva, Marcio Ajudarte Lopes

**Affiliations:** 1 al Diagnosis Department, Piracicaba Dental School, University of Campinas, Piracicaba, Brazil

## Abstract

**Background:**

This retrospective study investigates the clinicopathological features and outcomes of young and elderly patients diagnosed with lip squamous cell carcinoma (LSCC).

**Material and Methods:**

Data from LSCC patients from Dr. Luiz Antonio Hospital in Natal, Brazil (2000-2015) were analyzed, grouping individuals below 40 and above 60 years old. Demographics, lifestyle habits, clinicopathologic characteristics, and treatment outcomes were examined using descriptive statistics, Chi-square and Fisher's tests, and Kaplan-Meier survival analysis.

**Results:**

A total of 47 patients was analyzed, being 20 younger and 27 older, finding significant age-related differences (*p* = < 0.0001). Although in both groups the tumor was more common in males, older patients had a higher rate of females (29.6%) (*p*=0.0358) and smoking (70.4%) (*p* = 0.0043) and underwent more modalities of treatments (*p* = 0.0027). There were no significant differences in the other analyzed clinicopathologic factors, and survival rates did not differ significantly, though younger patients showed slightly better survival metrics in univariate analysis.

**Conclusions:**

LSCC exhibits some distinct clinicopathological features across different age groups, with significant differences in treatment modalities and progression rates. Age-specific approaches may be required to optimize treatment outcomes.

** Key words:**Squamous cell carcinoma, lip, young, survival, prognosis.

## Introduction

Lip squamous cell carcinoma (LSCC) is a malignant neoplasm that originates from the epithelium of the vermilion border of the lip, which marks the transition between the skin of the face and the oral cavity (1,2). The lower lip is the most commonly affected area, accounting for about 90% of LSCC cases, while approximately 7% of LSCCs occur on the upper lip and 3% at the labial commissure (1,2). Similar to basal cell carcinoma, squamous cell carcinoma and skin melanoma, LSCC is primarily caused by mutations resulting from ultraviolet (UV) radiation from the sun (3). Additional contributing factors include smoking, alcohol consumption, genetic predisposition, and immunocompromised status (1,2,4). In Brazil, LSCC poses a significant public health issue due to the country's tropical climate, which results in high levels of UV radiation exposure (4).

The population predominantly affected by LSCC comprises males in their sixth and seventh decades of life, characterized by fair skin phenotypes and a reduced capacity for tanning (1,2,5). LSCC exhibits an encouragingly high overall 5-year survival rate of 80 to 90%, attributed in part to low cervical lymph node metastatic rates of 2% to 5% (5). Despite these favorable statistics, a specific subset of patients with LSCC demonstrates poor prognostic outcomes due to delayed cervical metastases, which result in a diminished 5-year survival rate ranging from 40% to 50% (5,6).

The recommended therapeutic approach for LSCC involves initial surgical resection, which may or may not be complemented by radiotherapy depending on the pathological characteristics of the LSCC (2,5). The surgical protocol dictates excision of the primary tumor together with a margin of apparently healthy tissue, with a suggested margin of 1 cm for LSCC (2,7). Additionally, a neck dissection is indicated in cases where metastatic lymph nodes are suspected (5).

To date, the scientific literature lacks specific studies on LSCC in patients under 40 years of age; however, it is established that this demographic constitutes less than 4% of all oral malignancies (8). Sporadic research indicates that in younger populations, the tongue and lip are the predominant subsites for oral cancer, whereas in elderly patients, the tongue and floor of the mouth are more frequently affected (8,9). The risk factors for the increased incidence of LSCC in young patients remain unclear. It is hypothesized that LSCC in young patients may represents a distinct biological entity, differing from that observed in elderly patients (9,10).

This study aims to compare the clinicopathologic characteristics of LSCC between young (<40 years) and elderly patients (>60 years), focusing on lifestyle habits, treatment modalities, and survival rates.

## Material and Methods

- Sample selection and data collection

This retrospective study involved analyzing clinicopathological data retrieved from patient records at Dr. Luiz Antonio Hospital in Natal, Brazil. The study cohort was determined by convenience, considering the uncommon incidence of LSCC. It consisted of individuals diagnosed with LSCC and treated between 2000 and 2015, who were either below 40 years or above 60 years of age at diagnosis. Exclusion criteria included patients aged 41 to 59 years and patients with tumors in other anatomical regions than the upper and lower lip. Data extracted for analysis encompassed demographic characteristics, lifestyle habits, clinical presentation, tumor features, treatment modalities, disease progression, and survival outcomes.

- Survival endpoints

Survival endpoints were categorized into three groups: Overall Survival, which represented the duration from treatment initiation to the latest follow-up; Disease-Specific Survival, indicating the period from treatment initiation to OSCC-related death or latest follow-up for surviving patients; and Disease-Free Survival, delineating the time from treatment initiation to the first recurrence diagnosis or latest follow-up for recurrence-free patients.

- Statistical analysis

The statistical analysis included descriptive and quantitative approaches. Chi-square (more than 15 samples) and Fisher's exact (fewer than 15 samples) tests were utilized to analyze the categorical data and to explore associations between clinicopathological parameters and patients’ status. Survival analysis involved constructing Kaplan-Meier curves, with between-group differences assessed using the log-rank univariate test to identify potential prognostic factors. Statistical analyses were performed using SPSS (IBM®, New York, USA) Version 22.0, with a significance level set at *p-value* ≤ 0.05.

## Results

- Demographic and clinical characteristics

A total of 47 patients were included in the study, being 20 younger than 40 years and 27 older than 60 years. The mean age among young patients was 36.5 years (range 23-39), while it was 72 years (range 61-92) in the elderly group. Gender distribution revealed that males accounted for 19 patients (95% in young and 70.4% in elderly patients) in both age groups, while females constituted one patient (5%) in the young group and eight patients (29.6%) in the older group, being this difference statistically significant (*p*=0.0358). Smoking was observed more frequently in the elderly group (19 patients, 70.4%) compared to only in four patients (20%) in the young group (*p*=0.0043). Alcohol intake, were similar between groups corresponding to three (15%) young patients and six (22.2%) elderly patients (*p*=0.3566). Both groups exhibited more patients with an evolution time ≤ 6 months, corresponding to 11 patients (55%) and 14 patients (52.9%), respectively (*p*=0.9769). In terms of tumor location, the lower lip was predominantly affected in both young (18 patients; 90%) and elderly (27 patients; 100%) groups (*p*=0.0966) (Table 1).

- Tumor characteristics and treatment modalities

More young patients were classified as T1 (16 cases, 80%) compared to elderly patients (13 cases, 48.1%). On the other hand, more elderly patients were classified as T2 (11 cases, 40.7%) compared to young patients (3 cases, 15%), but without statistically significant differences between groups (*p*=0.1518). As for lymph node metastasis, although young patients had more cases classified as N0 (19 patients, 95%) compared to elderly patients (23 cases, 85.2%), the difference was not statistically significant (*p*=0.3501). In terms of distant metastasis, in both groups the majority of the patients were classified as M0, being 95% in young patients and 88.9% in elderly patients (*p*=0.7412). Regarding histological grade, most of the patients had tumors classified as grade II, being 85% and 81.5% of the young and elderly patients, respectively (*p*=0.8732). In terms of treatment, most of the young patients underwent only surgery (19 patients, 95%). On the other hand, elderly patients, underwent more combination of treatments and with difference statistically significant between groups (*p*=0.0027) (Table 1).

- Histological features of the surgical specimen and clinical outcomes

Surgical margins and perineural invasion were similar between groups (*p*=0.5022 and *p*=0.3798). The events of local recurrence, regional recurrence, distant metastasis and second primary tumor were also similar between groups (*p*=0.1072, *p*=0.5549, *p*=0.3984 and *p*=0.9103).

In terms of status, most of both groups were alive, being 18 patients in the young group (90%) and 24 patients in the elderly group (88.9%) (*p*=0.2338). Regarding disease-free survival, the mean was 28 months (range 0-137) for young patients and 18 months (range 0-184) for elderly patients *p*=0.7469) (Table 1).

- Univariate survival analysis

**Figure 1:**
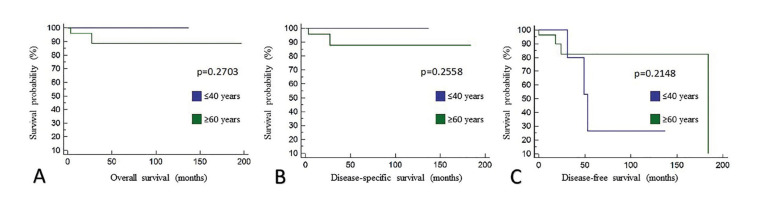
Survival analysis. A) Overall 5-year survival (*p*=0.2703); B) Disease-specific 5-year survival (*p*=0.2558); C) Disease-free survival at 5 years (*p*=0.2148).

The univariate analysis of survival (Kaplan-Meier) was conducted to compare the survival rates between patients ≤40 years of age and patients ≥60 years of age. The overall 5-year survival, the disease-specific 5-year survival and disease-free survival at 5 years were similar between groups (*p*=0.2703, *p*=0.2558 and *p*=0.2148) (Fig. 1).

## Discussion

In recent years, there has been a noTable increase incidence of oral squamous cell carcinoma (OSCC) among young patients, prompting several studies to explore the biological behavior of these tumors within this group (9,10). It is important to note that definitions of "young" patients vary; some studies classify individuals under the age of 45 as young, while others consider the cutoff to be under 40 years old (11-13). Consequently, the age criteria for categorizing patients as young are not uniform across the literature (11,12). For the purposes of the current study, individuals up to the age of 40 were classified as young.

Gathering information on lip cancer presents challenges due to its location in a transitional zone between the skin and the mucosa (14). Moreover, the definitions of the oral cavity's boundaries differ across studies (15,16), with some researchers considering the lips as part of the oral cavity while others do not (17). Owing to this disagreement, the lip was categorized as an extraoral structure in the current study.

LSCC is one of the most common subsites of OSCC, accounting for 30% of these cases and generally exhibiting a favorable prognosis. The incidence rate is approximately 12 per 100,000 per year in North America (5). About 90% of tumors occur on the lower lip, 7% on the upper lip, and 3% at the oral commissure (1,2). These findings are consistent with our study, where the majority of tumors were diagnosed on the lower lip. In addition, male individuals are mostly affected (1,5), in accordance with the results observed in the research, which demonstrated statistically significant association between males and LSCC. The most common subsite for OSCC in both young and elderly patients is the tongue (8). Although LSCC is also frequently observed in elderly, it is not a common subsite in young individuals, corresponding to just 5% of all OSCC (5,8).

There is an increasing incidence of OSCC in younger patients, who are believed to have an etiology distinct from that of elderly patients, due to their reduced exposure to common risk factors such as tobacco and alcohol (9-11,18,19). Although LSCC is generally categorized as oral cancers, its etiology is considered to be distinct (10,11,20). LSCC is typically caused by chronic sun exposure, whereas OSCC is more commonly associated with tobacco use, with or without alcohol consumption (20). Interestingly, our analysis revealed that smoking was associated with the presence of LSCC in patients over 60 years of age. It may be explained by the synergism between smoking metabolites and the sun exposure, increasing the possibility of LSCC. Elderly individuals present more exposure to tabacco use, which may also be associated with this result. Moreover, differences in smoking habits between young and elderly patients can be attributed to changes in societal attitudes, peer influence, and health education over time.

In regards the evolution-time, the current study showed a lower evolution time in months in both age groups. Interestingly, some studies explained that elderly patients exhibit repulsion to oncology treatments, which causes a more advance disease stage (5,6). Considering the young patients, it has been described that the biological behavior of OSCC in young patients presents an aggressive characteristic (11,12), which may also be associated with our results.

The clinical tumor-node-metastasis (TNM) stage is crucial and helps in the evaluation of the aggressiveness of LSCC, leading in the determination of the most appropriate treatment (21). In the present study, the majority of cases in both groups were diagnosed at early stages (stages I and II). However, when considering more advanced stages of the disease, elderly patients were predominantly affected. This observation aligns with prior studies, which have demonstrated that tumors at stages III and IV, especially those affecting the labial commissure, are typically more locally aggressive and prone to recurrence, with a high incidence of cervical metastases (21,22). Notably, none of these relationships were observed in the current research, indicating an accordance from previous findings.

In LSCC, the presence of metastasis is a strong predictor of poor outcomes, with a survival rate of only 40 to 50% for patients with lymph node metastasis over five years (5,6). Fortunately, the incidence of lymph node metastasis in LSCC is relatively low, ranging between 5% and 37% of cases (6). Additionally, the TNM classifications were not associated with statistically significant results in our study, aligning with the literature on LSCC (5,6). Importantly, our study found that the rate of regional metastasis was low in both groups, reinforcing the observations from existing research on the relatively lower metastatic risk in LSCC.

In LSCC, treatment options include surgery or radiotherapy (22). In our study, young patients primarily underwent isolated surgical removal of LSCC, indicating a preference for this treatment in younger demographics. Conversely, elderly patients more frequently received a combination of treatments, a difference that was statistically significant. Treatment outcomes vary due to physiological differences, duration of smoking, and adherence to treatment protocols. Younger patients often benefit from better health, more robust immune systems, and greater access to healthcare resources. Furthermore, local, regional, and distant recurrences, as well as the occurrence of second primary tumors, were more common, although without statistical differences, among patients over 60 years of age, highlighting an increased complexity in managing LSCC in older populations.

It is well established that lip cancer generally has better survival rates than intraoral cancers (23,24). However, it remains unclear whether significant differences in prognosis exist between young and elderly patients with lip cancer. Our statistical analysis found no substantial differences between these age groups, suggesting that age may not significantly influence the prognosis of lip cancer. This finding aligns with previous research (18,19). The lack of observed differences may be due to the impact of comorbid health conditions in elderly patients, which could affect their overall prognosis and potentially influence the analysis of this parameter (10-12).

This study presents several limitations, including the fact that the sample was retrieved from just one center, which may influence the sample size. Additionally, the retrospective nature of the data collection introduces potential biases such as selection bias, since only patients with available records were included. Recall bias may also be present, as historical data depends on the accuracy and completeness of the medical records.

## Conclusions

This study underscore the necessity of integrating age-specific considerations into the management of LSCC. The statistically significant variations in gender distribution and smoking habits between younger and older patients imply that distinct, tailored approaches to treatment and prevention are warranted. Moreover, the observed differences in treatment modalities across age groups suggest that personalized treatment plans may significantly enhance patient outcomes.

Future research should prioritize prospective studies to validate these findings and elucidate the molecular mechanisms driving LSCC in different age demographics. Investigating the interplay between genetic predispositions and environmental factors will deepen our understanding of the disease's progression and may pave the way for developing targeted therapies. Additionally, examining the long-term outcomes of various treatment strategies will be crucial in establishing evidence-based best practices for managing LSCC across diverse age groups.

## Figures and Tables

**Table 1:** Distribution of clinicopathological parameters in each group of young and elderly patients.

Variables		≤40	≥60	p-value
		N (%)	N (%)	
Age	Range	23-39	61-92	-
	Mean	36.5	72.0	< 0.0001
Sex	Male	19 (95%)	19 (70.4%)	0.0358
	Female	1 (5%)	8 (29.6%)	
Smoking	Absent	9 (45%)	5 (18.5%)	0.0043
	Present	4 (20%)	19 (70.4%)	
	NR	7 (35%)	3 (11.1%)	-
Alcohol intake	Absent	5 (25%)	4 (14.8%)	0.3566
	Present	3 (15%)	6 (22.2%)	
	NR	12 (60%)	17 (63.0%)	-
Evolution time (months)	≤ 6	11 (55%)	14 (52.9%)	0.9769
	> 6	8 (40%)	10 (37.0%)	
	NR	1 (5%)	3 (11.1%)	-
Primary site	Upper lip	2 (10%)	0 (0%)	0.0966
	Lower lip	18 (90%)	27 (100%)	
Tumor size (T)	T1	16 (80%)	13 (48.1%)	0.1518
	T2	3 (15%)	11 (40.7%)	
	T3	1 (5%)	2 (7.4%)	
	T4	0 (0%)	1 (3.7%)	
	TX	0 (0%)	0 (0%)	-
Lymph node metastasis (N)	N0	19 (95%)	23 (85.2%)	0.3501
	N1	0 (0%)	2 (7.4%)	
	N2	0 (0%)	1 (3.7%)	
	N3	0 (0%)	1 (3.7%)	
	NX	1 (5%)	0 (0%)	-
Distant metastasis (M)	M0	19 (95%)	24 (88.9%)	0.7412
	M1	0 (0%)	0 (0%)	
	MX	1 (5%)	3 (11.1%)	-
Histological grade	I	2 (10%)	4 (14.8%)	0.8732
	II	17 (85%)	22 (81.5%)	
	III	1 (5%)	1 (3.7%)	
Treatment	Radical surgery	19 (95%)	13 (48.1%)	0.0027
	Radical surgery+RT	1 (5%)	7 (25.9%)	
	Radical surgery+RT+CT	0 (0%)	7 (25.9%)	
Surgical margins	Free	17 (85%)	23 (85.2%)	-
	Compromised	1 (5%)	3 (11.1%)	0.5022
	NR	2 (10%)	4 (14.8%)
Perineural invasion	Absent	6 (30%)	22 (81.5%)	0.3798
	Present	0 (0%)	3 (11.1%)	
	NR	14 (70%)	2 (7.4%)	-
Local recurrence	Absent	4 (20%)	23 (85.2%)	0.1072
	Present	3 (15%)	4 (14.8%)	
	NR	13 (65%)	0 (0%)	-
Regional recurrence	Absent	1 (5%)	19 (70.4%)	0.5549
	Present	1 (5%)	8 (29.6%)	
	NR	18 (90%)	0 (0%)	-
Distant recurrence	Absent	9 (45%)	24 (88.9%)	0.3984
	Present	0 (0%)	2 (7.4%)	
	NR	11 (55%)	1 (3.7%)	-
Second primary site	Absent	5 (25%)	23 (85.2%)	0.9103
	Present	1 (5%)	4 (14.8%)	
	NR	14 (70%)	0 (0%)	-
Status	Alive	18 (90%)	24 (88.9%)	0.2338
	Dead	0 (0%)	2 (7.4%)	
	NR	2 (10%)	1 (3.7%)	-
Disease-free survival	Range	0-137	0-184	-
	Mean	28	18	0.7469

Abbreviations: CT - chemotherapy; RT - radiotherapy.
